# Supplementary material for: Association between oral microbiome and seven types of cancers in East Asian population: a two-sample Mendelian randomization analysis
Source: Front Mol Biosci. 2023 Nov 21;10:1327893. doi: 10.3389/fmolb.2023.1327893 (PMC10702768; doi:10.3389/fmolb.2023.1327893)
Supplement: Supplementary file 1 [file DataSheet1.ZIP › Supplementary Materials/MR plots of saliva/saliva═╝/Prostate cancer/pheno.2411_to_prostate cancer_forest.pdf]

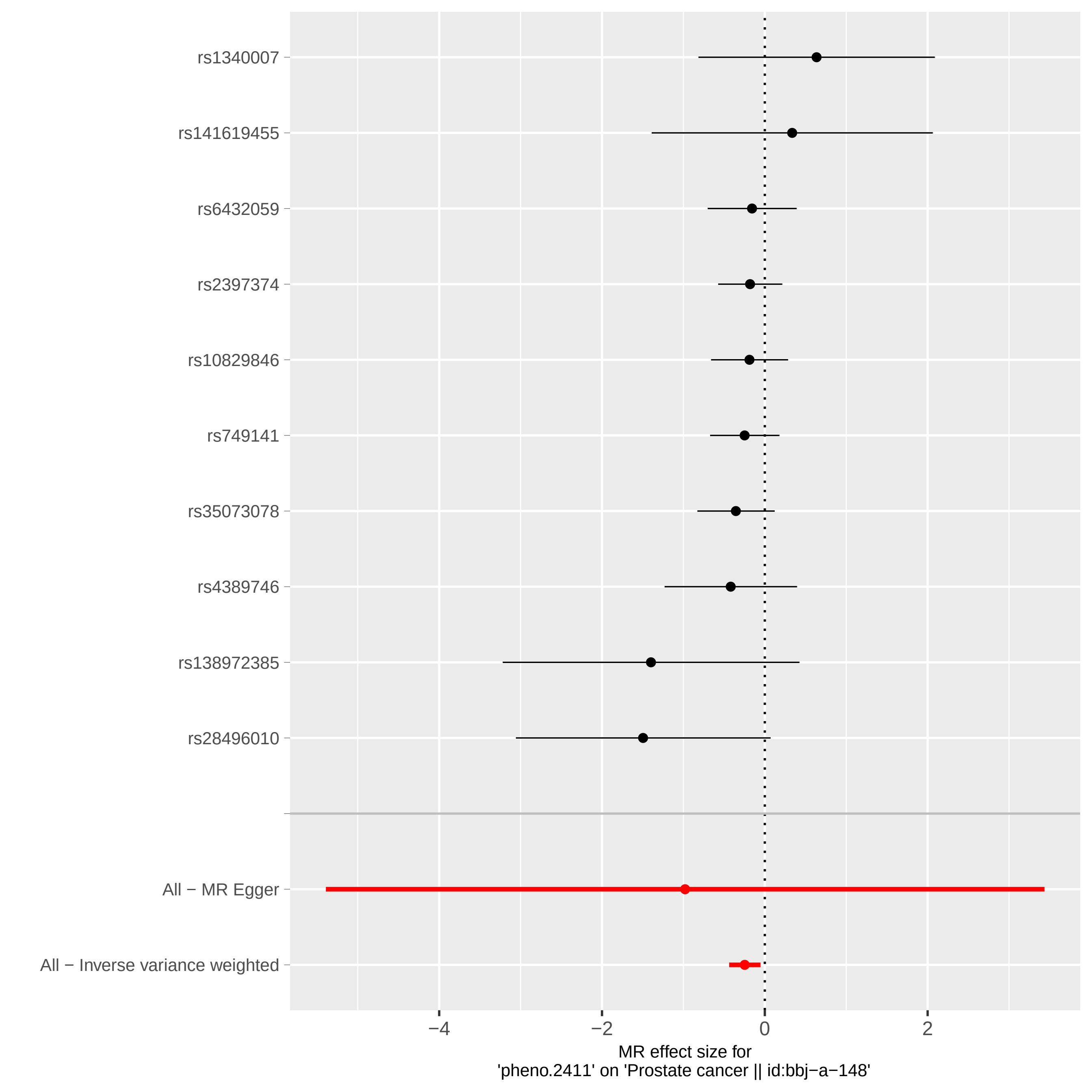
rs1340007
rs141619455
rs6432059
rs2397374
rs10829846
rs749141
rs35073078
rs4389746
rs138972385
rs28496010
All − MR Egger
All − Inverse variance weighted
−4
−2
0
2
MR effect size for
'pheno.2411' on 'Prostate cancer || id:bbj−a−148'
